# UHPLC/MS-MS Analysis of Six Neonicotinoids in Honey by Modified QuEChERS: Method Development, Validation, and Uncertainty Measurement

**DOI:** 10.1155/2013/863904

**Published:** 2013-03-31

**Authors:** Michele Proietto Galeano, Monica Scordino, Leonardo Sabatino, Valentina Pantò, Giovanni Morabito, Elena Chiappara, Pasqualino Traulo, Giacomo Gagliano

**Affiliations:** Dipartimento dell'Ispettorato Centrale della Tutela della Qualità e della Repressione Frodi dei Prodotti Agroalimentari (ICQRF), Laboratory of Catania, Ministero delle Politiche Agricole Alimentari e Forestali (MIPAAF), Via A. Volta 19, 95122 Catania, Italy

## Abstract

Rapid and reliable multiresidue analytical methods were developed and validated for the determination of 6 neonicotinoids pesticides (acetamiprid, clothianidin, imidacloprid, nitenpyram, thiacloprid, and thiamethoxam) in honey. A modified QuEChERS method has allowed a very rapid and efficient single-step extraction, while the detection was performed by UHPLC/MS-MS. The recovery studies were carried out by spiking the samples at two concentration levels (10 and 40 *μ*g/kg). The methods were subjected to a thorough validation procedure. The mean recovery was in the range of 75 to 114% with repeatability below 20%. The limits of detection were below 2.5 *μ*g/kg, while the limits of quantification did not exceed 4.0 *μ*g/kg. The total uncertainty was evaluated taking the main independent uncertainty sources under consideration. The expanded uncertainty did not exceed 49% for the 10 *μ*g/kg concentration level and was in the range of 16–19% for the 40 *μ*g/kg fortification level.

## 1. Introduction

Neonicotinoids are a relatively new class of insecticides that share a common mode of action that affect the central nervous system of insects, resulting in paralysis and death [1]. They possess either a nitromethylene, nitroimine, or cyanoimine group [2]. They include acetamiprid, clothianidin, imidacloprid, nitenpyram, thiacloprid, and thiamethoxam. Studies suggested that neonicotinoids residues can accumulate in pollen and nectar of treated plants and represent a potential risk to pollinators [3]. Therefore, neonicotinic pesticides may play a role in recent pollinator declines. The Honey Italian Observatory stated that in 2008 more than half of Italian hives, and that 600,000 of a total of 1,100,000 have been put out of production for the depopulation of entire apiaries. The honey production in 2008 fell by 50% reduced to 7,000 tons. One result might be expected given that the previous year, the European Food Safety Authority (EFSA) stated that the bee die-off had hit the 50% bee population, compared to the annual average of 15%.

Neonicotinoids can also be persistent in the environment and, when used as seed treatments, translocate to residues in pollen and nectar of treated plants. The potential for these residues to affect bees and other pollinators remains uncertain. Despite these uncertainties, neonicotinoids are beginning to dominate the market place because of their high systemicity, the broad spectrum of action, and the reduced dose. In light of these findings, the Italian Ministry of Agriculture has asked the Ministry of Health to suspend action. The Ministry of Health, after consultation with the Pesticides Committee, issued the ministerial decree of September 17, 2008 that stated the precautionary suspension of the authorized use for the seeds tanning of plant protection products containing the active substances clothianidin, thiamethoxam, imidacloprid, and fipronil [4]. On June 25, 2012, a decree of the Ministry of Health extended to January 31, 2013 stating the neonicotinoids suspension for seeds treatment [5]. Similar measures have already been taken by other European states.

Recently, many researchers detected these insecticides in honey bees, honey, soil, pollen, and treated seeds for agriculture [6–12]. Measurement of pesticide residues in different matrices involves two basic steps, namely, sample preparation (extraction and clean up) and instrumental analysis. Ideally, a sample preparation should be rapid, simple, cheap, and environment friendly and provide clean extracts. After extraction, clean up is the most important process for multiresidue analysis. QuEChERS (Quick Easy Cheap Effective Rugged Safe) technique, which was developed between 2000 and 2002 and first reported in 2003 [13], is a fast and complete extraction and clean up procedure and also employs the use of dispersive-solid phase extraction (d-SPE) for sample clean up.

In this paper, we report a rapid modified QuEChERS method for multiresidue analysis for 6 neonicotinoids (acetamiprid, clothianidin, imidacloprid, nitenpyram, thiacloprid, and thiamethoxam) in honey with good selectivity, sensitivity, and cost effectiveness. In order to demonstrate the suitability of the method for routine regulatory purposes, the method was validated and the statistical parameters are discussed.

## 2. Materials and Methods

### 2.1. Reagents and Standards

The certified analytical standards of all the 6 pesticides (acetamiprid, clothianidin, imidacloprid, nitenpyram, thiacloprid, and thiamethoxam) and internal standard Tris(1-chloro-2-propyl)phosphate (TCPP) were purchased from Ultra Scientific (Bologna, Italy) (100.0 ± 0.5 *μ*g/mL each) in acetonitrile. All the solvents and chemicals used in the study were of analytical reagent (AR) grade, ethanol was supplied by Romil (Milan, Italy), and formic acid, ammonium formiate, and acetonitrile were by Carlo Erba (Milan, Italy). Distilled water was purified at 18.2 MΩ with a MilliQ ULTRA (Millipore, Vimodrone (MI), Italy) purification system. A mixture of dispersive SPE Citrate Extraction Tube Supelco (4 g magnesium sulphate, 1 g sodium chloride, 0.5 g sodium citrate dibasic sesquihydrate, and 1 g sodium citrate tribasic dihydrate) was used, supplied by Sigma-Aldrich (Milan, Italy).

### 2.2. Instrumentation

Ultra high-performance liquid chromatography UHPLC-MS/MS (Thermo Scientific, TSQ Quantum Access Max) equipped with Thermo hypersilgold column (50 mm × 2.1 mm, 1.9 *μ*m) was used for quantification of neonicotinoids. The flow rate was 400 *μ*L/min, the column temperature 30°C, and the injection loop volume 5 *μ*L. A binary gradient of 0.05% HCOOH and HCOONH_4_ 2 mM in water (A) and 0.05% HCOOH and HCOONH_4_ 2 mM in CH_3_OH (B) was employed. The mobile-phase gradient was programmed as follows: 0 min, 10% B; 7 min, 95% B; 8 min, 95% B; 9 min, 10% B; and 10 min, 10% B. Mass spectral analyses were performed using an LC-TSQ Quantum Access Max operating in the positive ion mode using a h-ESI interface. The electrospray ionization (ESI) needle spray voltage was 4000. The heated capillary was 270°C. Flush volume was 700 *μ*L and Collision Gas Pressure was 1.3 mTorr. The neonicotinoids and the internal standard TCPP were detected in MS/MS conditions, programming the chromatographic run in SRM mode (selected reaction monitoring) as reported in Table 1. Preliminary tunings were carried out with continuous introduction of a dilute solution of certified standards. Flow rate of syringe pump infusion of 5 *μ*L/min and the voltages on the lenses were optimized in TSQ Tune Master (Excalibur software).

### 2.3. Reference Solution

The standard mix solution at 5 *μ*g/mL of standard pesticides was diluted by transferring 500 *μ*L (100.0 ± 0.5 *μ*g/mL) into a volumetric flask (10 mL, Class A certified). 

The standard mix solution at 1 *μ*g/mL of standard pesticides was diluted by transferring 100 *μ*L (100.0 ± 0.5 *μ*g/mL) into a volumetric flask (10 mL, Class A certified). 

The standard mix solution at 0.1 *μ*g/mL of standard pesticides was diluted by transferring 200 *μ*L of solution at 5 *μ*g/mL into a volumetric flask (10 mL, Class A certified). All mix solutions are making up at volume with acetonitrile. Stock solutions stored at −18°C were stable for at least 3 months.

### 2.4. Method Validation

#### 2.4.1. Specificity

The specificity of the analytical method for neonicotinoids detection was confirmed by obtaining positive results from honey containing the analyte, coupled with negative results from samples which do not contain it (negative controls). The matrix effect was assessed by preparing pesticide standards in blank matrix extracted from untreated honey. The matrix extracts were analyzed before spiking to confirm the absence of the test pesticides in them. 

#### 2.4.2. Linearity

The quantification of pesticide was based on a six-point matrix-matched calibration graph by plotting the detector response (SRM area ratio with respect to internal standard TCPP) against concentration of the calibration standards within the range 1–50 *μ*g/L making three replicates for each concentration. A linear regression of six calibration points for each component was used to determine the relationship with the analyte concentrations calculated for each component on the basis of their occurrence in the reference material. The regression equations with slope, *y*-intercept, and coefficient of correlation (*r* ^2^) were evaluated for acetamiprid, clothianidin, imidacloprid, nitenpyram, thiacloprid, and thiamethoxam. Statistical test (Mandel and residual analysis with normal distribution of the calibration points) were performed to prove the linearity of regression lines.

#### 2.4.3. Limit of Detection (LOD) and Limit of Quantification (LOQ)

The LOD and LOQ were determined by signal-to-noise approach [14]. The noise and signal are measured experimentally on the chromatogram printout. LOQ was estimated by the response of method noise level by approximately ten and LOD is, therefore, 3.3-fold lower.

#### 2.4.4. Method Accuracy (Recovery) and Precision (Repeatability)

Method recovery studies were performed at two spiking concentration levels (10 *μ*g/kg and 40 *μ*g/kg). The sample matrix was prepared by homogenizing a series of different honeys in order to develop a highly specific method. The samples were prepared by weighing 5.0  ±  0.5 g of honey spiked in 50 mL tube (Meus srl, Piove di Sacco (PD), Italy). These sample tubes were vortexed (Velp, Usmate (MB), Italy) for 30 seconds after adding 10 mL of water and 10 mL of acetonitrile, in order to homogenize and fluidize the sample, and 50 *μ*L of Tris(1-chloro-2-propyl)phosphate (TCPP) at 50 mg/L. In each tube was added a mixture of salts (4 g magnesium sulphate, 1 g sodium chloride, 0.5 g sodium citrate dibasic sesquihydrate, and 1 g sodium citrate tribasic dihydrate). The extract was stirred for 1 minute in vortex, in order to maximize the distribution of the analytes in the organic phase. The samples were centrifuged at 3000 rpm for 5 minutes and the supernatant was filtered at 0.45 *μ*m PTFE filters (VWR, Milan, Italy). The extract was analyzed by UHPLC-MS/MS, making 6 replicates for each concentration. The average percentage of recovery and the relative standard deviation (RSD, repeatability) were evaluated.

#### 2.4.5. Determination of Uncertainties

Combined uncertainty in estimation was determined for all the neonicotinoids at the two fortification levels studied (10 and 40 *μ*g/kg) as the statistical procedure of the EURACHEM/CITAC Guide CG 4 [15]. Individual sources of uncertainty were taken into account as described below.

*Uncertainty of Analytical Standard Solutions*. As the uncertainty of standard concentration declared in the supplier's certificate was given without any confidence level, rectangular distribution was assumed for calculating standard uncertainty

(1) $$\begin{array}{c} U1=\frac{\;u\left(x\right)/C\left(x\right)}{\surd 3}, \end{array}$$

where *u*(*x*) represents the uncertainty value given in the certificate and *C*(*x*) the concentration of the standard solution.

*Uncertainty of Weighing*. The relative uncertainty due to honey weighing was calculated using normal distribution given by

(2) $$\begin{array}{c} U2=\frac{\left(0.00005\right)}{Wi}, \end{array}$$

where *Wi* is the weight of the sample, and 0.00005 is the value of uncertainty of the balance at 95% confidence level as reported in the certificate. 

*Uncertainty of Calibration Linearity. *Uncertainty associated with the calibration curve, was calculated according to

(3) $$\begin{array}{c} U3=\;{\left(\frac{s}{b_{1}}\right)\;\left(\left\{\frac{1}{p}\right\}+\left\{\frac{1}{n}\right\}+\;\left\{\frac{{\left(c_{0}-c^{\prime }\right)}^{2}}{s_{xx}}\right\}\right)}^{1/2}, \end{array}$$

where *s* is the standard deviation of the residuals of the calibration curve, *b*_1_ is the slope of the calibration curve, *p* is the number of measurements of the unknown, *n* is the number of points used to form the calibration curve, *c*_0_ is the calculated concentration of the analyte from the calibration curve, *c*′ is the arithmetic mean of the concentrations of the standards used to make the calibration curve, and *s*_*xx*_ is calculated as given in

(4) $$\begin{array}{c} s_{xx}=\sum {\left(cj-\;c^{\prime }\right)}^{2}, \end{array}$$

where *j* = 1, 2,…, *n*. *cj* is the concentration of each calibration standard used to build up the calibration curve.

*Uncertainty Associated with Precision.* In the present study, the random errors of extraction, clean up, and UHPLC analyses steps were approximated by standard deviations which were calculated from repeated determinations of analytes expressed as repeatability. The precision was calculated according to

(5) $$\begin{array}{c} U4\;=\frac{s}{\left(\surd n\times x\right)}, \end{array}$$

where *s* is the standard deviation of the results obtained from the recovery study, *n* is the number of assays and *x* is the mean value of the concentration recovered.

*Uncertainty of Volume.* The volume of the solution is subject to 3 sources of uncertainty: calibration, repeatability, and temperature effects. (a) Calibration: the uncertainty in the certified internal volume of the flask and of the pipettes. For example, the manufacturer gives a volume of 10.00 ± 0.02 mL (*V* ± *a*) for the flask, when measured at a temperature of 20°C. Because the value of the uncertainty is given without a confidence level or distribution information, an assumption is necessary. In this work, the standard uncertainty is calculated by assuming a triangular distribution according to

(6) $$\begin{array}{c} U5\;=\frac{\left(a/\surd 3\right)}{V}\;. \end{array}$$

In the same way, the volumes of the pipettes used to prepare the solutions at different levels are calculated by assuming a triangular distribution. The contributions due to the dilution operations performed for each concentration level are calculated separately and combined to give the standard uncertainty of the volume. (b) Repeatability: the uncertainty due to variations in filling is considered in the repeatability experiments. (c) Temperature: the temperatures of the flask and solution differ from the temperature at which the volume of the flask was calibrated. According to the manufacturer, the flask was calibrated at a temperature of 20°C, whereas the laboratory temperature varies by ±2°C. The uncertainty from this effect can be calculated from the estimate of the temperature range and the coefficient of the volume expansion. In the case of acetonitrile as a solvent, this effect is negligible.

The combined uncertainty (*U*) was calculated as = *x*[(*U*1^2^ + *U*2^2^ + *U*3^2^ + *U*4^2^)^1/2^], where *Cx* is the mean neonicotinoids concentration, and reported as expanded uncertainty (2*U*) which is twice the value of the combined uncertainty at 95% confidence level.

## 3. Results and Discussion

### 3.1. Method Development

In order to identify the major species produced in collisional experimental fragmentation of MS/MS analysis, a mass characterization study was firstly performed for direct infusion of each investigated neonicotinoids. Mass scans in positive ions mode were performed with h-ESI source ionization; all investigated molecules showed a good fragmentation. The collision energy was modulated from 5 to 50 of instrumental maximum to obtain the better fragmentation pattern. The ESI spectrum is characterized by the parent ion [M + H]^+^ for all molecules. The neutral losses of NO_2_ and/or HCl were observed for clothianidin, imidacloprid, nitenpyram, and thiamethoxam. The fragment at *m*/*z* 126, corresponding to [C_6_H_5_-OCl]^+^ was a characteristic for acetamiprid, nitenpyram, and thiacloprid (Table 1). The discussed SRM data were in agreement with what reported by Sabatino et al. [10] and Ferrer et al. [16].

**Figure 1:**
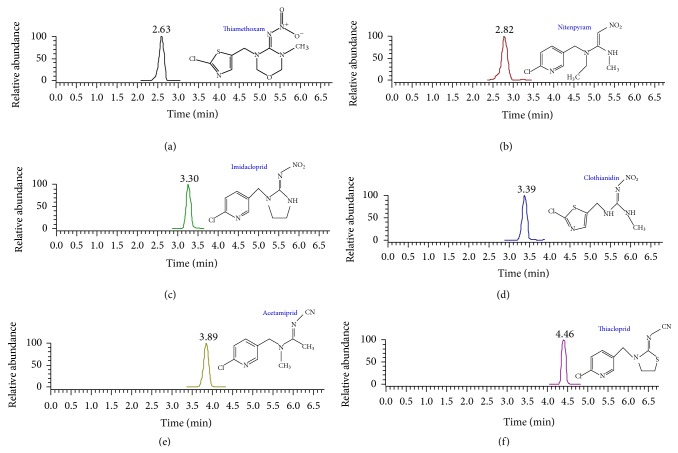
Representative UHPLC/MS-MS chromatogram of studied neonicotinoids for matrix-matched standards 40 *μ*g/kg.

The chromatographic method has been developed on the results of preliminary studies carried out on matrix-fortified standards. Different solvents were used for the chromatographic separation and several chromatographic separations were evaluated. The best results were obtained using an elution gradient starting with a binary gradient of 0.05% HCOOH and HCOONH_4_ 2 mM in water and 0.05% HCOOH and HCOONH_4_ 2 mM in CH_3_OH combined with the Thermo hypersil GOLD 50 × 2.1 mm (1.9 *μ*m i.d.) column. Under the described chromatographic conditions, the studied molecules were resolved in less than 5 minutes (Figure 1) and well recognizable on the basis of *m*/*z* signals, and good sensitivities were obtained; each analyte showed a typical mass spectrum profile previously identified by direct infusion. 

The concept of a single extraction and dilution of the extracts was chosen in this study to achieve good results in the shortest time. In 2011, Tanner and Czerwenka [11] applied two steps of purification with d-SPE applying the QuEChERS methodology to the honey. Our protocol eliminated the second purification step, limiting the extraction to the use of d-SPE citrate extraction tube and reducing times and costs of analyses. Nevertheless, results were satisfactory in terms of statistical parameters, the selectivity for the analytes of interest, and reduction of the matrix effect (see paragraph below). This protocol permitted to analyze a high number of samples per day and is, therefore, suitable for a routine application in control laboratories. The proposed analytical protocol is currently applied in ICQRF Catania laboratory in the frame of Italian Ministry quality control investigation.

### 3.2. Method Validation

Analytical parameters of the proposed method were evaluated according to the criteria given in Section 2. Results are reported in Table 2.

#### 3.2.1. Specificity

The specificity of the method toward the studied analytes was good. No interferences due to matrixes were found. Hence, no further time-consuming concentration/cleanup pretreatments were required. 

#### 3.2.2. Linearity of Calibration Curve

The linearity of each pesticide was established by plotting UHPLC response area ratio versus concentration. The analytes showed linear behavior in the studied concentration range of 1–50 *μ*g/L. The correlation coefficient (*r* ^2^) was found to be ≥0.995 for all pesticides. 

#### 3.2.3. LOD and LOQ

LOD and LOQ were estimated as the lowest concentrations of pesticide injected that yielded a signal/noise ratio of 3 and 10, respectively. LOQs evaluation showed the lowest value 0.10 *μ*g/kg for thiacloprid to the higher value of 4.00 *μ*g/kg for nitenpyram. The LOQs attained in the proposed method fit with maximum residue limits (MRLs) of 10 *μ*g/kg for nonallowed pesticides [17].

#### 3.2.4. Recovery and Precision

The single-step extraction method adopted for honey samples provided satisfactory recovery which ranged from 75% (nitepyram) to 114% (imidacloprid) for the fortification level of 10 *μ*g/kg and from 92 (thiacloprid) to 109% (imidacloprid) for the fortification level of 40 *μ*g/kg. The precision of the method was good, not exceeding a coefficient of variation of 12%, with the exception of nitenpyram at the lowest fortification level. These data are in agreement with the criteria of document no. SANCO/12495/2011, that recommend general recovery limits of 70–120% within laboratory repeatability ≤20% [18]. Therefore, the method could be considered sufficiently accurate and precise for the purpose. 

#### 3.2.5. Uncertainty of Measurement

The study of uncertainty was performed at 2 concentration levels (10 and 40 *μ*g/kg), identifying and studying the most important parameters that determined the uncertainty of the analytical method. The parameters selected were point calibration, standard solution, weigh, volume, and precision; their contributions to method uncertainty were calculated as indicated in the experimental section. The different contributions of uncertainty for each concentration level, together with the relative combined standard uncertainty, are shown in Tables 3 and 4 for each neonicotinoid. Results showed that the contribution to uncertainty due to the dilution operations and the standard purities was constant for each concentration level and for each analyte. The same value of uncertainty concerning the amount of weighed sample was used for each level and for all pesticides because the quantity of analyzed sample did not change among the experiments; moreover, this contribution could be considered negligible. The uncertainty associated with repeatability has a moderate contribution to the expanded uncertainties, showing the higher value for nitenpyram, thiamethoxam, and clothianidin. The 10 *μ*g/kg level showed the uncertainty of calibration point as the main constituent of total uncertainty, followed by the volume contribution. On the contrary, the volume uncertainty was the major source to total uncertainty at the 40 *μ*g/kg level, while the uncertainty of repeatability and calibration point had approximately similar values.

When the uncertainty of the result is reported, the combined standard uncertainty is multiplied with a so-called coverage factor, yielding an expanded uncertainty. A factor *k* = 2 was used because of the resemblance of the expanded uncertainty to a 95% confidence interval. The document no. SANCO/12495/2011 recommended a default expanded uncertainty of 50% to be used by regulatory authorities in cases of enforcement decisions (MRL exceedances) [18]. Our results showed a relative uncertainty (*U*%) ranging from 21 (thiamethoxam) to 49% (acetamiprid) at levels of 10 *μ*g/kg. Lower values were obtained for the 40 *μ*g/kg level. At this level, all pesticides had *U*% ranging from 16 to 19%.

## Figures and Tables

**Table 1:** UHPLC-MS/MS fragmentation of studied neonicotinoids.

MW	Pesticide	Precursor ion (*m*/*z*)	Product ions (*m*/*z*)	Collision energy (eV)
			90.3	31
222	Acetamiprid	223.0	99.2	34
			126.2	19

			113.2	25
249	Clothianidin	250.0	132.1	16
			169.2	12

			90.3	36
252	Thiacloprid	253.0	99.2	37
			126.2	21

255	Imidacloprid	256.0	175.2	17
			209.1	14

			126.2	27
270	Nitenpyram	271.1	225.2	9
			237.2	21

			181.2	20
291	Thiamethoxam	292.0	210.2	7
			211.2	10

**Table 2:** Method validation results.

Compound	R_t_ (min)	Linearity range (*μ*g/L)	r^2^	LOQ (µg/kg)	Recovery % (10 *μ*g/kg)^†^	Recovery % (40 *μ*g/kg)^†^
Thiamethoxam	2.63	1–50	0.999	0.50	101 ± 11	100 ± 12
Nitenpyram	2.82	1–50	0.998	4.00	75 ± 20	97 ± 9
Imidacloprid	3.30	1–50	0.998	2.80	114 ± 3	109 ± 7
Clothianidin	3.39	1–50	0.999	3.20	111 ± 8	105 ± 9
Acetamiprid	3.89	1–50	0.995	0.12	107 ± 5	105 ± 6
Thiacloprid	4.46	1–50	0.997	0.10	89 ± 6	92 ± 2

^†^Mean value of six determinations; relative standard deviations (precision) in parenthesis.

**Table 3:** Results of individual and combined uncertainties for each pesticide calculated at 10 *μ*g/kg concentration level.

Compound	Standard solution	Weighting	Calibration curve	Precision	Dilution operations	Combined uncertainty	Expanded uncertainty	Relative expanded uncertainty
	*U*1	*U*2	*U*3	*U*4	*U*5	*U*	2*U*	*U*%
Thiamethoxam	0.003	0.00001	0.064	0.044	0.070	1.1	2.2	21
Nitenpyram	0.003	0.00001	0.164	0.082	0.070	1.5	3.0	39
Imidacloprid	0.003	0.00001	0.161	0.011	0.070	2.0	4.0	35
Clothianidin	0.003	0.00001	0.089	0.033	0.070	1.3	2.6	23
Acetamiprid	0.003	0.00001	0.232	0.019	0.070	2.6	5.2	49
Thiacloprid	0.003	0.00001	0.220	0.024	0.070	2.1	4.2	46

**Table 4:** Results of individual and combined uncertainties for each pesticide calculated at 40 *μ*g/kg concentration level.

Compound	Standard solution	Weighting	Calibration curve	Precision	Dilution operations	Combined uncertainty	Expanded uncertainty	Relative expanded uncertainty
	*U*1	*U*2	*U*3	*U*4	*U*5	*U*	2*U*	*U*%
Thiamethoxam	0.003	0.00001	0.017	0.040	0.070	3.3	6.6	16
Nitenpyram	0.003	0.00001	0.036	0.032	0.070	3.3	6.6	16
Imidacloprid	0.003	0.00001	0.040	0.029	0.070	3.7	7.4	17
Clothianidin	0.003	0.00001	0.023	0.037	0.070	3.5	7.0	16
Acetamiprid	0.003	0.00001	0.061	0.023	0.070	4.0	8.0	19
Thiacloprid	0.003	0.00001	0.054	0.009	0.070	3.3	6.6	17
